# Liver Androgen Receptor Knockout Improved High-fat Diet Induced Glucose Dysregulation in Female Mice But Not Male Mice

**DOI:** 10.1210/jendso/bvae021

**Published:** 2024-02-09

**Authors:** Adjoa Osei-Ntansah, Trinitee Oliver, Taylor Lofton, Claire Falzarano, Kiana Carr, Ruthe Huang, Andre Wilson, Ella Damaser, Guyton Harvey, Md Ahasanur Rahman, Stanley Andrisse

**Affiliations:** Department of Physiology and Biophysics, Howard University College of Medicine, Washington, DC 20059, USA; Department of Physiology and Biophysics, Howard University College of Medicine, Washington, DC 20059, USA; Department of Physiology and Biophysics, Howard University College of Medicine, Washington, DC 20059, USA; Department of Physiology and Biophysics, Howard University College of Medicine, Washington, DC 20059, USA; Department of Physiology and Biophysics, Howard University College of Medicine, Washington, DC 20059, USA; From Prison Cells To PhD, Baltimore, MD 21224, USA; Department of Physiology and Biophysics, Howard University College of Medicine, Washington, DC 20059, USA; Department of Physiology and Biophysics, Howard University College of Medicine, Washington, DC 20059, USA; Department of Physiology and Biophysics, Howard University College of Medicine, Washington, DC 20059, USA; Department of Physiology and Biophysics, Howard University College of Medicine, Washington, DC 20059, USA; Department of Physiology and Biophysics, Howard University College of Medicine, Washington, DC 20059, USA

**Keywords:** polycystic ovary syndrome, metabolic syndrome, androgen receptor, insulin resistance, metabolism

## Abstract

Previous research has indicated that liver androgen receptors may play a role in modulating disease. This study aims to investigate the pathophysiology of high-fat diet (HFD) induced dysglycemia in male and female liver androgen receptor knockout (LivARKO) mice. We performed metabolic tests on LivARKO female and male mice fed a HFD or a control diet (from Research Diets Inc.) during months 1 or 2 after starting the diet. Additionally, we performed Western blot and quantitative real-time PCR analysis on the livers of the mice to examine intermediates in the insulin signaling pathway. LivARKO-HFD female mice displayed no difference in glucose tolerance compared to female LivARKO-Control (Con) mice, whereas in wild-type female mice, HFD impaired glucose tolerance (IGT). Our data suggests that starting at 1 month, LivARKO may be protecting female mice from HFD-induced metabolic dysfunction. LivARKO-HFD female mice displayed significantly worse insulin sensitivity at 15 minutes compared to LivARKO-Con female mice, but, strangely, LivARKO-HFD female mice had significantly better insulin sensitivity at 60 and 90 minutes compared to LivARKO-Con female mice. Despite protecting against IGT, LivARKO did not protect against HFD-induced hyperinsulinemia in female mice. In contrast to females, male LivARKO-HFD mice displayed impaired glucose tolerance compared to male LivARKO-Con mice. Thus, LivARKO is not protective against HFD-induced glucose metabolic dysfunction in male mice. Lastly, LivARKO-HFD female mice maintained hepatic insulin sensitivity whereas LivARKO-HFD male mice displayed hepatic insulin resistance. These findings suggest that LivARKO delayed the onset of HFD-induced dysglycemia in female mice.

Hepatic insulin resistance is linked with many metabolic diseases such as polycystic ovary syndrome [1], type 2 diabetes [2], and nonalcoholic fatty liver disease [3]. Many hepatic insulin resistance models have been suggested [4], but a considerable amount of data supports the participation of diacylglycerol (DAG) buildup and protein kinase C epsilon (PKCε) stimulation in damaging hepatic insulin signaling [5]. Abnormal amounts of lipids in the liver relate to hepatic insulin resistance independent of obesity and visceral adiposity [6].

In high-fat diet (HFD) rodents, elevated hepatic DAG content stimulated PKCε, the main protein kinase C (PKC) isoform in liver [7], resulting in lowered stimulation of insulin receptor (IR) [8] via serine phosphorylation of IR and/or insulin receptor substrate-1 or -2. Decreasing PKCε levels shielded rodents from fat-induced hepatic insulin resistance and maintained IR function, despite hepatic DAG and triglyceride content being unaltered [8]. Likewise, *Prkce*−/− mice were shielded from fat-induced insulin resistance after 1 week of HFD, in spite of elevated hepatic lipid content [9]. These data suggest a model for hepatic insulin resistance where the buildup of liver DAG stimulates PKCε and weakens IR function and establishes that PKCε stimulation is needed for fat-induced hepatic insulin resistance.

Liver androgen receptor (AR) knockout mice (LivARKO) have been developed previously by Lin et al in 2008 [10]. HFD male but not female LivARKO mice developed hepatic steatosis and insulin resistance, and aging male LivARKO mice fed chow exhibited moderate dysglycemia. Thus, deleting AR in the livers of male mice was detrimental in HFD and aging. However, the Lin et al 2008 study or any other study since, aside from my preliminary work, has examined female LivARKO mice in an hyperandrogenemia (HA) model [11] or HFD model (here).

Here we sought to investigate the effect of hepatic deletion of AR in HFD-induced insulin-resistant male and female mice. AR has traditionally been thought of as only having nuclear function as a transcription factor. However, several recent studies [11, 12] have shown that AR directly interacts with cytosolic signaling pathways [13]. My laboratory's previous work has shown that HA-induced hepatic insulin resistance is driven by cytosolic AR binding to and reducing the activity of PI3K, leading to decreased p-AKT [11, 12]. Here, we propose that hepatic AR will *not* be primarily involved in disrupting insulin signaling in an HFD-induced state of hepatic insulin resistance, but it may display some crosstalk.

## Materials and Methods

### Generation of LivARKO Mice

Floxed AR (Jackson Laboratories, B6.129S1-Artm2.1Reb/J) and albCre (Jackson Laboratories) mice were maintained in our laboratory. We generated LivARKO mice (ARfl/fl; Albcre+/−) by mating heterozygous female (ARfl/wt; Albcre+/−) with homozygous male (ARfl/y; Albcre-/−) mice. As controls, we used littermates with genotype ARfl/wt; Albcre-/−, and/or ARfl/fl, Albcre-/−. We called these mice wild-type (WT) throughout the study. We are aware they are not true WT, but we assessed them, and they did not display whole-body glucose metabolic changes from true WT (data not shown). Genomic DNA isolation and primers to detect the AR gene and AlbCre were described previously and provided later [14]. All procedures were performed with the approval of the Howard University Animal Care and Use Committee.

### Genotyping of Mouse Genomic DNA

*Floxed-AR*: AAA ATG CCT CCT TTT GAC CA (Forward), AAG ATG ACA GTC CCC ACG AG (Reverse) https://www.jax.org/strain/018450

*Alb Cre*: TGC AAA CAT CAC ATG CAC AC (WT Forward), TTG GCC CCT TAC CAT AAC TG (Common), GAA GCA GAA GCT TAG GAA GAT GG (Mutant Forward) https://www.jax.org/strain/003574

### Experimental Design

C57BL/6 male and female WT mice (as described earlier) and LivARKO mice were placed on 1 of 2 diets from 4 weeks to 12 weeks of age:

1. Control (Con) [Research Diets Inc, RDI D12450J; protein (20%), carbs (70%; corn starch 50%, sucrose-maltodextrin 20%, fructose 0%), fat 10% (lard)] or
2. HFD (RDI D12492; protein (20%), carbs (20%; corn starch 0%, sucrose-maltodextrin 20%, fructose 0%), fat 60% (lard)].

Some studies have shown that starting HFD between 6 and 10 weeks of age is the most optimal for investigating metabolic outcomes [15]. However, other studies have shown that starting a HFD at 4 weeks of age is feasible for studying metabolic changes during the peripubertal age [16]. We are interested in studying metabolic derangements starting during the peripubertal age so as to be comparable with our previous findings [17] and other findings using this age in mice [18] and translatable to humans of pubertal age [19]. Glucose, insulin, and pyruvate tolerance tests (PTTs) and glucose-stimulated insulin secretion (GSIS) tests were performed 1 and 5, 2 and 6, 3 and 7, and 4 and 8 post-treatment, respectively.

### Metabolic Testing

Mice fasted for 7-hours received intraperitoneal injections of (1) 2 g/kg body weight (BW) glucose [glucose tolerance tests (GTT)], (2) 0.3 units/kg BW insulin [insulin tolerance tests (ITT)] (Lilly, Indianapolis, IN), or (3) 2 g/kg BW pyruvate (PTTs) (Sigma-Aldrich, St. Louis, MO), and tail blood was obtained 0, 15, 30, 60, 90, and 120 minutes after injection to determine blood glucose levels using a One Touch Ultra glucometer (Life Scan, Inc., Milpitas, CA). Tail blood was obtained for all time points by 1 single small less than 1 mm cut at the end of the tail. This causes very minimal pain as described by the National Resource Council's Guide for Care and Use of Lab Animals (8th edition). For the GSIS tests, blood samples were obtained at time 0, at 15 minutes, and at 30 minutes after 2 g/kg BW glucose intraperitoneal injection to 7-hour fasted mice, and sera were separated by centrifugation. GSIS levels were measured using an ELISA Insulin Assay (Sigma-Aldrich). We obtained 30 μL of blood for serum analysis. Intraperitoneal injections as opposed to oral administration was used to avoid changes due to manipulation of incretins in the gut [20]. Fasting times were used according to standards in the field [21].

### Plasma Estradiol

Blood was collected by cardiac puncture at sacrifice using EDTA-treated tubes. The plasma was obtained after centrifugation and stored at −80 ˚ for further analysis. Plasma estradiol levels were measured via an estradiol kit (KGE014, Novus Biologicals) according to the manufacturer's protocol. The plasma was diluted 1:5 for Con diet and HFD samples.

### RNA Isolation and qPCR

Frozen tissue was homogenized with a tissue homogenizer (Qiagen, Chatsworth, CA), and the total RNA was extracted with QIAzol and purified using miRNeasy Mini Kit (Qiagen) according to the manufacture's protocol. Quantitative and qualitative analyses of the RNA were performed with BioTek Epoch Microplate Spectrophotometer (Agilent Technologies, Palo Alto, CA). The samples displayed RNA integrity >6.5. One microgram RNA underwent reverse transcription via an iScript cDNA synthesis kit (Bio-Rad). Quantitative PCR was performed using 10 nanogram of cDNA in the CFX Duet Touch System (Bio-Rad), with iTaq universal SYBR Green supermix (Bio-Rad). Samples were run in duplicates, and the relative gene expression was calculated as the mean per group using the ΔΔCt method, normalized to the mean of reference genes (*Actb*). Primer sequences are as follows.

### qRT-PCR Primers: Sense (5′ to 3′) and Antisense (5′ to 3′)

Estrogen receptor alpha (*Esr1*); Sense TCTGCCAAGGAGACTCGCTACT and Antisense GGTGCATTGGTTTGTAGCTGGAC

Phosphoenolpyruvate carboxykinase (*Pck1*); Sense AGCATTCAACGCCAGGTTC and Antisense CGAGTCTGTCAGTTCAATACCAA

Glucose 6 phosphatase (*G6p*); Sense GCTGTGATTGGAGACTGGCTCA and Antisense GTCCAGTCTCACAGGTTACAGG

Androgen receptor (*Ar*); Sense CGGAAGCTGAAGAAACTTGG and Antisense ATGGCTTCCAGGACATTCAG

Actin (*Actb*); Sense CATTGCTGACAGGATGCAGAAGG and Antisense TGCTGGAAGGTGGACAGTGAGG

### Protein Processing and Western Blots

Liver tissues from male and female mice fasted for 4 to 6 hours and injected with saline or 0.5 units/kg BW insulin (Lilly, Indianapolis, IN) 10 minutes before being sacrificed were extracted and analyzed for total protein content with the bicinchoninic acid protein assay. Proteins were prepared for Western blot analysis with separation via SDS-PAGE and transferred to nitrocellulose membranes. The nitrocellulose membranes were blocked then incubated with primary antibodies [AR, estrogen receptor (ER)α, p-ERα-S167, AKT, p-AKT-S473, GAPDH, actin; varying dilution; Table 1]. ERα and p-ERα-S167 were purchased from Cell Signaling Technologies, and all other antibodies were from Santa Cruz Biotechnology. After incubation with secondary antibodies (goat anti-mouse IgG or mouse anti-rabbit; 1:5000 dilution; Santa Cruz Biotechnology), enhanced chemiluminescence was used for detection. Densities were quantified via myImage Analysis software (Thermo Fisher Scientific) and analyzed by two-tailed t-tests with Prism software.

**Table 1.** Antibodies

Peptide/Protein target	Antibody ID (RRID)	Name of antibody	Antibody manufacturer, catalog no.	Animal in which antibody was raised; monoclonal or polyclonal	Dilution
Androgen receptor	AB_626671	AR (441)	Santa Cruz Biotechnology, sc-7305	Mouse, monoclonal	1:500
Estrogen receptor α	AB_2617128	ER alpha (D8H8)	Cell Signaling Technology, cat. no. 8644	Rabbit, monoclonal	1:1000
Phospho-estrogen receptor α (Ser167)	AB_2799660	p-ER-alpha S167 (D5W3Z)	Cell Signaling Technology, cat no. 64508	Rabbit, monoclonal	1:1000
Akt1/2/3 (5C10)	AB_1118808	Akt1/2/3 (5C10)	Santa Cruz Biotechnology, sc-81434	Mouse, monoclonal	1:1000
p-Akt1/2/3 (protein kinase B)	AB_2861344	p-AKT S473 (C-11)	Santa Cruz Biotechnology, sc-514032	Mouse, monoclonal	1:500
Glyceraldehyde 3-phosphate dehydrogenase	AB_10847862	GAPDH (g-9)	Santa Cruz Biotechnology, sc-365062	Mouse, monoclonal	1:1000
β-Actin	AB_626632	β-Actin (c4)	Santa Cruz Biotechnology, sc-47778	Mouse, monoclonal	1:1000
Goat anti-mouse IgG-HRP	AB_631736	Goat Anti-Mouse	Santa Cruz Biotechnology, sc-200	Goat, polyclonal	1:5000
Mouse anti-rabbit IgG-HRP	AB_628497	Mouse Anti-Rabbit	Santa Cruz Biotechnology, sc-2357	Mouse, polyclonal	1:5000
Estradiol Parameter Assay Kit	AB_2861157	Estradiol ELISA	R and D Systems (via Novus Biologicals) cat no. KGE014	Mouse, monoclonal	According to manufacturer
Rat/Mouse Insulin ELISA Kit	AB_2783856	Insulin ELISA	Millipore (via Sigma Aldrich) cat no. EZRMI-13K	Mouse, monoclonal	According to manufacturer

Abbreviations: HRP, horseradish peroxidase.

### Power Analysis

The “resource equation” method [22, 23] was used, and a biostatistician was consulted. All comparisons were sufficiently powered. Comparisons including male and female were slightly overpowered due to the need for more animals when only comparing 1 sex.

### Statistical Analysis

The groups were compared using a 2-way ANOVA (data reported as mean ± SEM); relationships between variables were determined by Pearson correlation, and sex proportions were compared with Fisher’s exact test. Data varying by time was analyzed by repeated-measures ANOVA (and reported as mean ± SEM), with post hoc tests by Bonferroni multiple comparisons test. Comparison of groups for continuous variables were performed using two-way ANOVA. Significance was set at *P* < .05.

## Results

### Verification of LivARKO and Experimental Design

**Figure 1.**
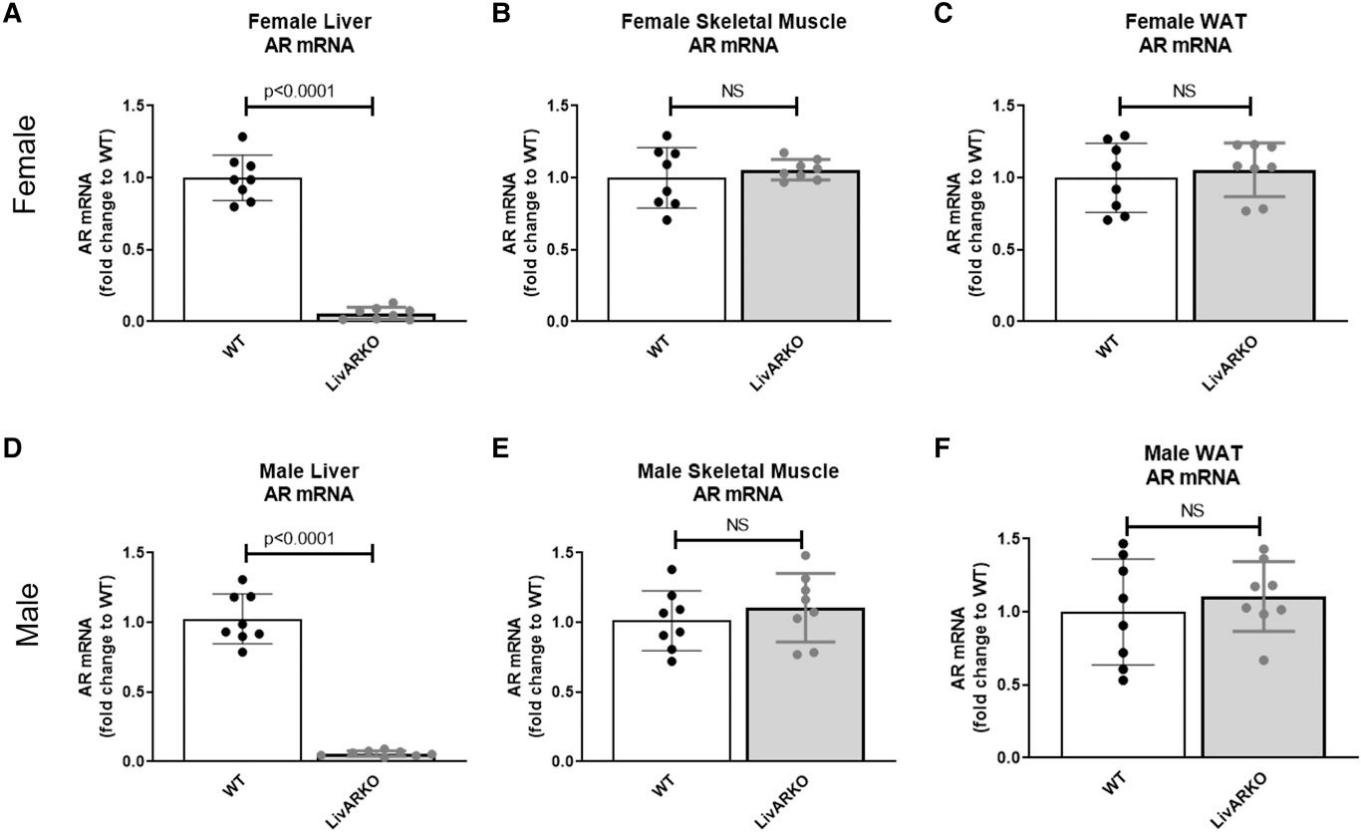
Verification of LivARKO via mRNA expression. Livers, skeletal muscles, and WAT were extracted from WT and LivARKO female mice after a 6-hour fast and underwent RNA processing and quantitative real-time PCR analysis for AR mRNA levels. (A) Female liver; (B) female skeletal muscles; (C) female WAT; (D) male liver; (E) male skeletal muscles; (F) male WAT (n = 8 per group). Abbreviations: AR, androgen receptor; LivARKO, liver androgen receptor knockout; WAT, white adipose tissues; WT, wild-type.

**Figure 2.**
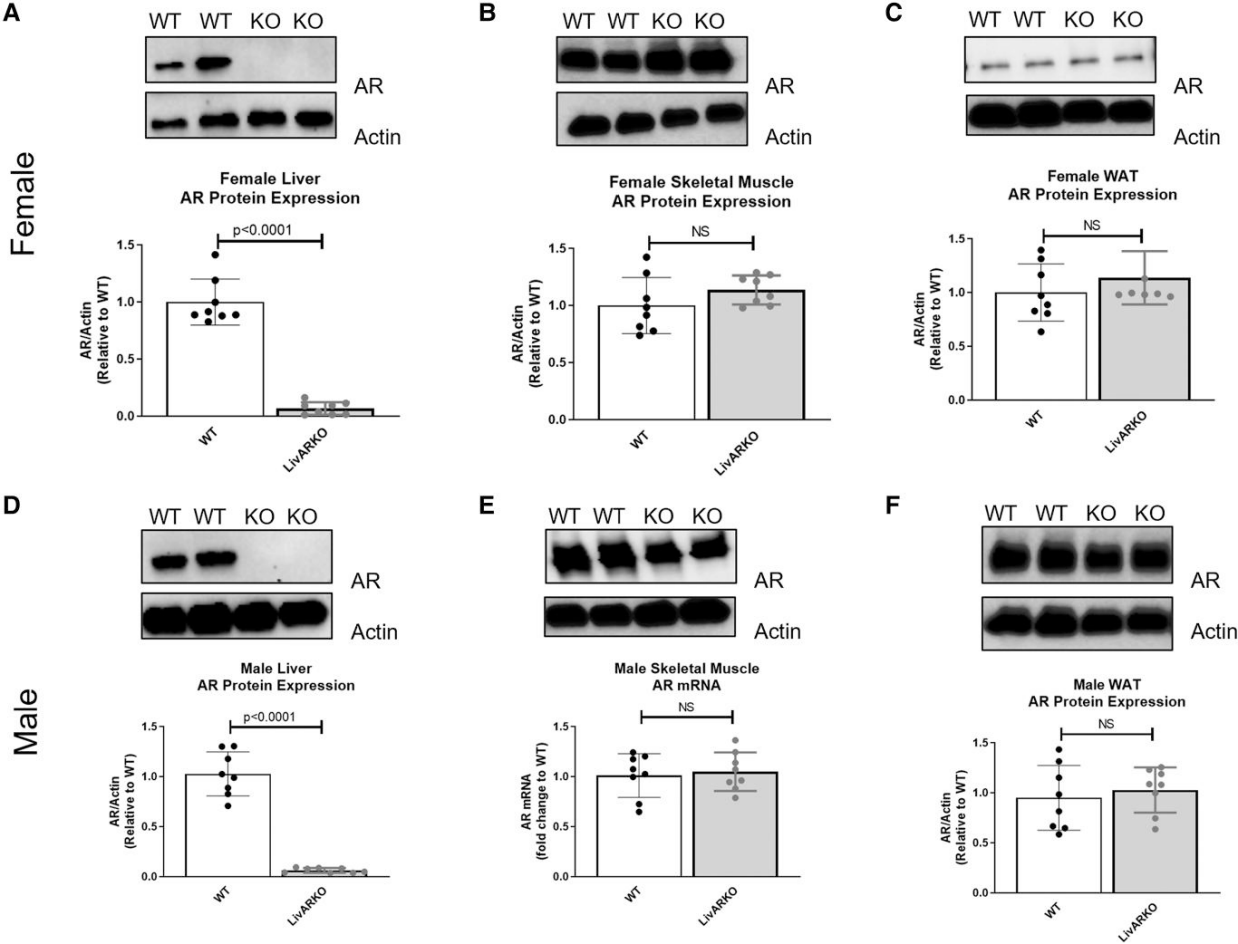
Verification of LivARKO via protein expression. Livers, skeletal muscles, and WAT were extracted from WT and LivARKO female mice after a 6-hour fast and underwent protein processing and Western analysis for AR protein levels. (A) Female liver; (B) female skeletal muscles; (C) female WAT; (D) male liver; (E) male skeletal muscles; (F) male WAT (n = 8 per group). Abbreviations: AR, androgen receptor; LivARKO, liver androgen receptor knockout; WAT, white adipose tissues; WT, wild-type.

**Figure 3.**
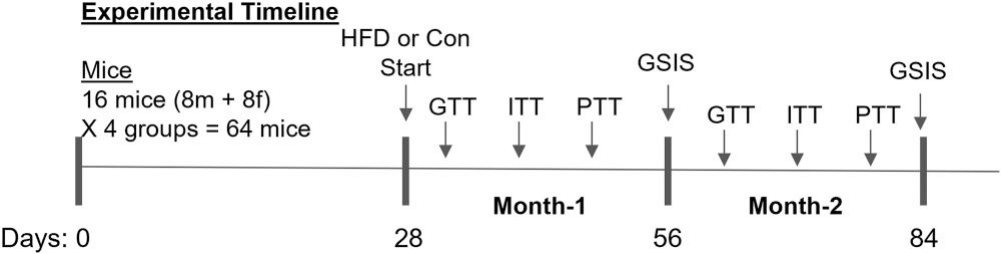
Experimental design. The experimental design for the 16 mice (8 m + 8 f) and 4 groups equaling 64 mice where glucose tolerance test, insulin tolerance test, pyruvate tolerance test, and glucose-stimulated insulin secretion were performed once per month following the implementation of a high-fat diet or a control diet at 28 days of age.

AR mRNA expression in the livers of LivARKO female mice was completely absent and significantly lower than that of WT female mice (Fig. 1A, *P* < .0001). AR mRNA expression in WT female mice compared to LivARKO female mice was the same in the skeletal muscles and the white adipose tissues (WAT), respectively (Fig. 1B and 1C). LivARKO male mice liver AR mRNA expression was completely absent and significantly lower than that of WT male mice (Fig. 1D, *P* < .0001), whereas LivARKO male mice AR mRNA expression was the same in the skeletal muscles and WAT, respectively, compared to WT male mice (Fig. 1E and 1F). To determine the AR protein expression in liver, skeletal muscles, and WAT in WT and LivARKO male and female mice, Western blot analysis was performed. AR protein expression was not present in male or female livers of LivARKO mice (Fig. 2A and 2D), and AR protein expression was the same in WT compared to LivARKO mice in skeletal muscles (Fig. 2B and 2E) and WAT (Fig. 2C and 2F). Figure 3 shows the experimental design where the mice were placed on a HFD at 28 days of age and subsequently underwent metabolic testing (GTT, ITT, PTT, and GSIS) once monthly for 2 months.

### Metabolic Tests in Females During Month 1

**Figure 4.**
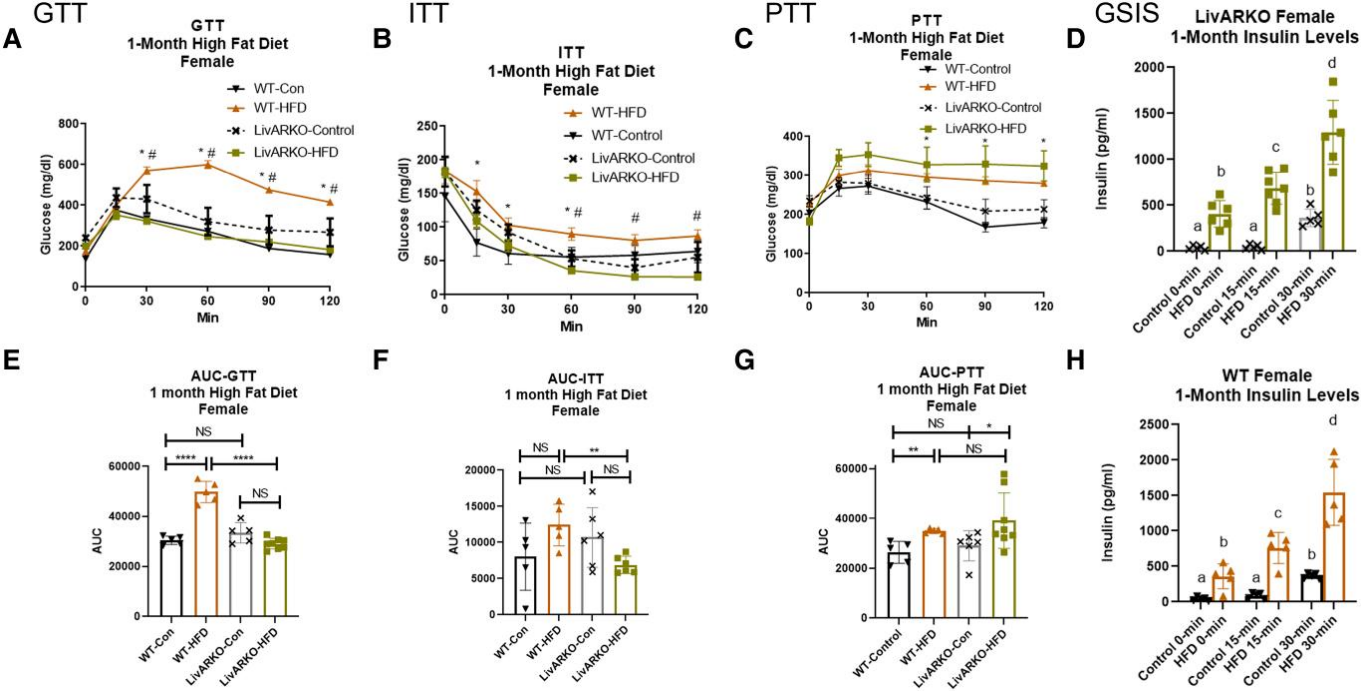
Metabolic tests in females at 1 month of HFD. During month 1 of HFD or Con diet feeding, WT and LivARKO female mice were subjected to (A) a 7-hour fasted, 2 g/kg BW intraperitoneal GTT (n = 5-8 per group), (B) a 7-hour fasted, 0.3 U/kg BW ITT (n = 5-8 per group); (C) a 7-hour fasted, 1 g/kg BW PTT (n = 5-8 per group); or (D) a 7-hour fasted GSIS analysis (LivARKO, n = 5-8 per group). (E) GTT AUC; (F) ITT AUC; (G) PTT AUC; (H) a 7-hour fasted GSIS analysis (WT, n = 5-8 per group). Difference between WT-Con and WT-HFD: *P < .05, **P < .001, ***P < .0001; difference between WT-HFD and LivARKO-HFD: #*P* < .05. Data are represented as mean ± SEM. Abbreviations: AUC, area under the curve; BW, body weight; Con, control; GSIS, glucose-stimulated insulin secretion; GTT, glucose tolerance test; HFD, high-fat diet; ITT, insulin tolerance test; LivARKO, liver androgen receptor knockout; PTT, pyruvate tolerance test; WT, wild-type.

At 1 month, WT-HFD female mice displayed impaired glucose tolerance (IGT) compared to WT female mice on a control diet (WT-Con) as seen by their significantly higher glucose levels during a GTT (Fig. 4A, *P* < .0001, [orange bar of the area under the curve (AUC), Fig. 4E, compared to the black bar]. Surprisingly, starting at 1 month, LivARKO completely prevented the HFD-induced IGT in female mice as 1-month LivARKO-HFD female mice displayed the same glucose levels as LivARKO female mice on a control diet [LivARKO-Con; Fig. 4A, green bar of AUC (Fig. 4E) compared to gray bar] and reduced glucose levels compared to WT-HFD female mice [Fig. 4A, *P* < .0001, green bar compared to orange bar of the AUC (Fig. 4E)]. At 1 month, WT-HFD female mice did not display impaired insulin sensitivity (IIS) compared to WT-Con female mice (Fig. 4B and 4F, orange compared to black bars). Similarly, at 1 month, LivARKO-HFD female mice did not display IIS compared to LivARKO-Con female mice (Fig. 4B and 4F, green compared to gray bars). However, LivARKO-HFD female mice displayed significantly enhanced insulin sensitivity (lowered glucose levels during the ITT) compared to WT-HFD female mice (Fig. 4B and 4F, *P* < .01, green compared to orange bars).

At 1 month, WT-HFD and LivARKO-HFD female mice displayed impaired pyruvate tolerance (IPT) compared to WT-Con and LivARKO-Con female mice, respectively (Fig. 4C and 4G, *P* < .01, orange compared to black bars, and *P* < .05, green compared to gray bars). However, LivARKO-HFD female mice did not display improvements in pyruvate tolerance in comparison to WT-HFD (Fig. 4C and 4G, green compared to orange bars). At 1 month, LivARKO-HFD and WT-HFD female mice exhibited hyperinsulinemia at fasting and at 15 and 30 minutes during the GSIS tests compared to controls, respectively (Fig. 4D and 4H, *P* < .05, orange to black bars and green to gray bars).

### Metabolic Tests in Males During Month 1

**Figure 5.**
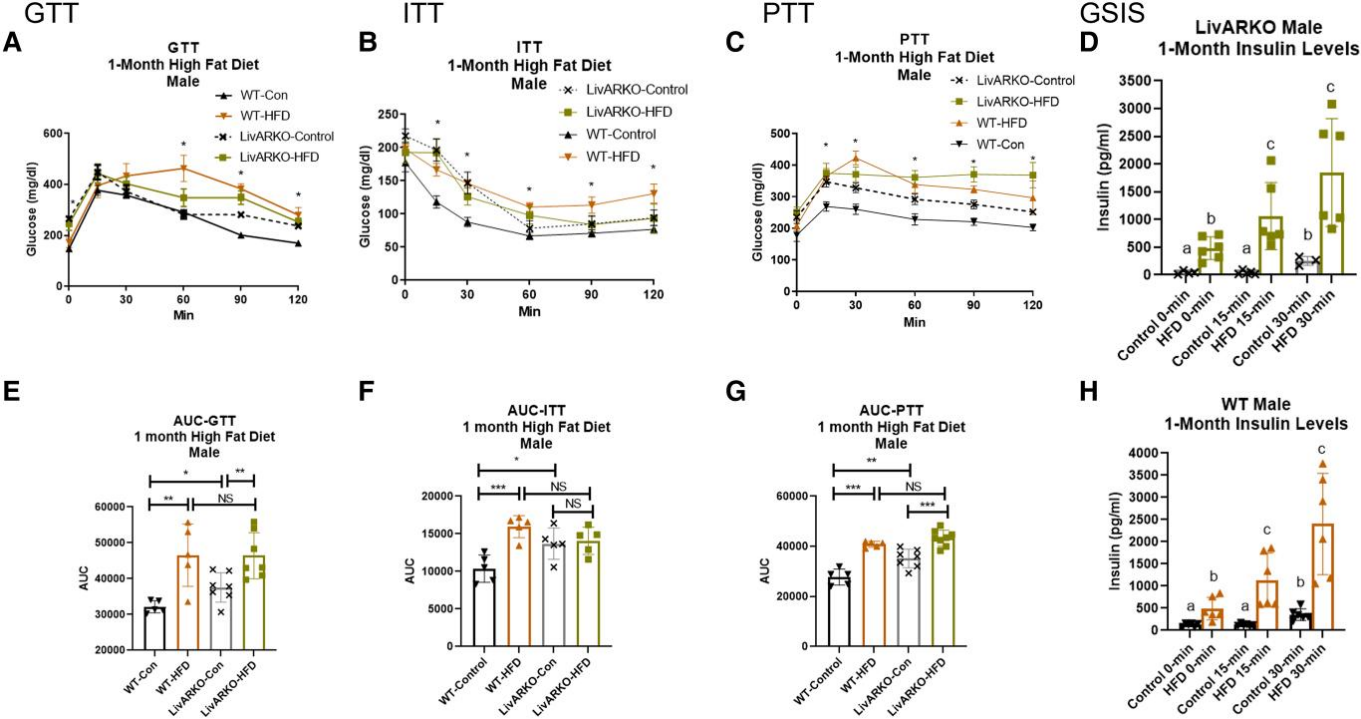
Metabolic tests in males at 1 month of HFD. During month 1 of HFD or Con diet feeding, WT and LivARKO male mice were subjected to (A) a 7-hour fasted, 2 g/kg BW intraperitoneal GTT (n = 5-8 per group), (B) a 7-hour fasted, 0.3 U/kg BW ITT (n = 5-8 per group); (C) a 7-hour fasted, 1 g/kg BW PTT (n = 5-8 per group); or (D) a 7-hour fasted GSIS analysis (LivARKO, n = 5-8 per group). (E) GTT AUC; (F) ITT AUC; (G) PTT AUC; (H) a 7-hour fasted GSIS analysis (WT, n = 5-8 per group). Difference between WT-Con and WT-HFD: *P < .05, **P < .001, ***P < .0001; difference between WT-HFD and LivARKO-HFD: #*P* < .05. Data are represented as mean ± SEM. Abbreviations: AUC, area under the curve; BW, body weight; Con, control; GSIS, glucose-stimulated insulin secretion; GTT, glucose tolerance test; HFD, high-fat diet; ITT, insulin tolerance test; LivARKO, liver androgen receptor knockout; PTT, pyruvate tolerance test; WT, wild-type.

At 1 month, WT-HFD male mice exhibited IGT compared to WT-Con male mice (Fig. 5A and 5E, *P* < .01, orange compared to black bar). Unlike in females, at one-month, LivARKO-HFD male mice still displayed IGT compared to LivARKO-Con male mice (Fig. 5A and 5E, green bar compared to gray bar) and similar glucose levels compared to WT-HFD male mice (Fig. 5A and 5E, *P* < .0001, green bar compared to orange bar). These data suggest that LivARKO prevented HFD-induced IGT in females but not in males, displaying a dimorphic effect. In contrast to females, at 1 month, WT-HFD male mice presented with IIS compared to WT-Con male mice (Fig. 5B and 5F, *P* < .001, orange compared to black bars). Interestingly, at 1 month, LivARKO-HFD male mice did not display IIS compared to LivARKO-Con male mice (Fig. 5B and 5F, green compared to gray bars). This appears to be because, unlike in females, LivARKO-Con displayed IIS compared to WT-Con male mice (Fig. 5B and 5F, *P* < .05, gray compared to black bars). LivARKO-HFD male mice displayed similar insulin sensitivity compared to WT-HFD male mice (Fig. 5B and 5F, green compared to orange bars).

At 1 month, WT-HFD and LivARKO-HFD male mice displayed IPT compared to WT-Con and LivARKO-Con male mice, respectively (Fig. 5C and 5G, *P* < .5, orange compared to black bars and green compared to gray bars). Similar to females, LivARKO-HFD male mice did not display improvements or derangements in pyruvate tolerance in comparison to WT-HFD (Fig. 5C and 5G, green compared to orange bars). However, LivARKO-Con was significantly higher than WT-Con in male mice (Fig. 5C and 5G, gray compared to black bars). At 1 month, LivARKO-HFD and WT-HFD male mice exhibited hyperinsulinemia at fasting, GSIS 15 minutes, and GSIS 30 minutes compared to controls, respectively (Fig. 5D and 5H, *P* < .05, orange to black bars and green to gray bars).

### Metabolic Tests in Females During Month 2

**Figure 6.**
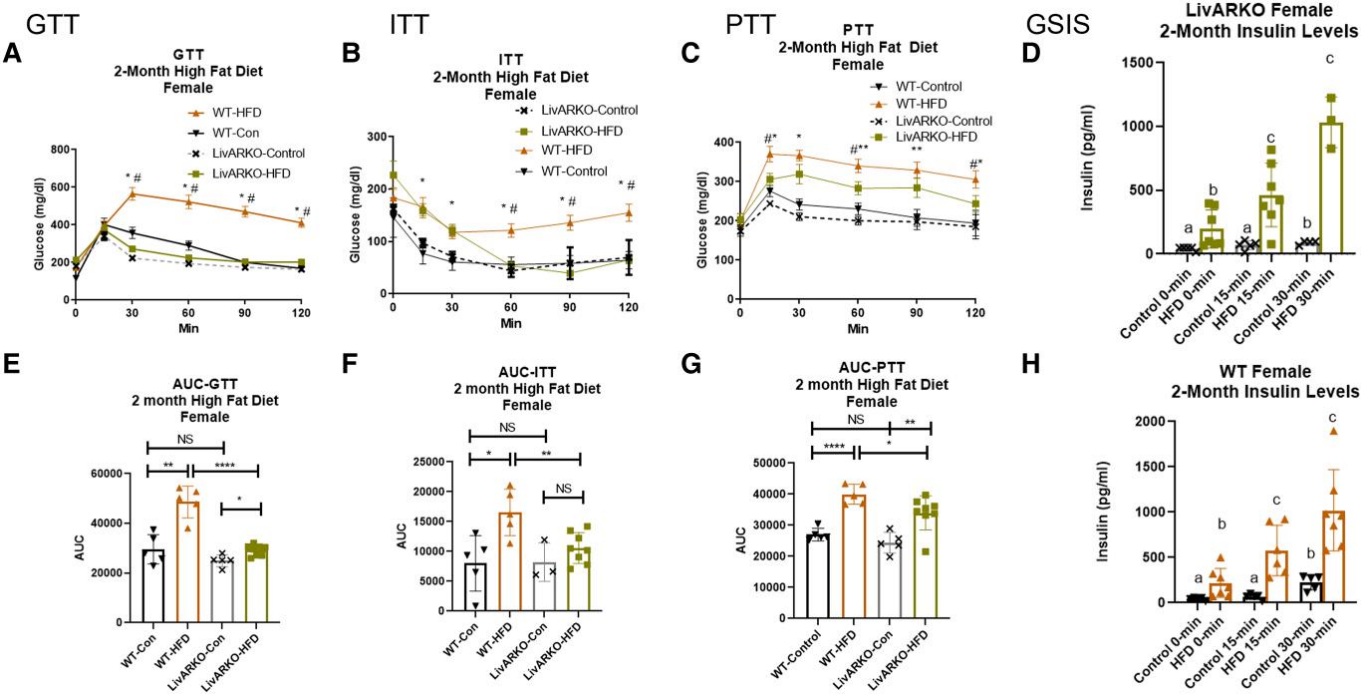
Metabolic tests in females at 2 months of HFD. During month 2 of HFD or Con diet feeding, WT and LivARKO female mice were subjected to (A) a 7-hour fasted, 2 g/kg BW intraperitoneal GTT (n = 5-8 per group), (B) a 7-hour fasted, 0.3 U/kg BW ITT (n = 5-8 per group); (C) a 7-hour fasted, 1 g/kg BW PTT (n = 5-8 per group); or (D) a 7-hour fasted GSIS analysis (LivARKO, n = 5-8 per group). (E) GTT AUC; (F) ITT AUC; (G) PTT AUC; (H) a 7-hour fasted GSIS analysis (WT, n = 5-8 per group). Difference between WT-Con and WT-HFD: *P < .05, **P < .001, ***P < .0001; difference between WT-HFD and LivARKO-HFD: #*P* < .05. Data are represented as mean ± SEM. Abbreviations: AUC, area under the curve; BW, body weight; Con, control; GSIS, glucose-stimulated insulin secretion; GTT, glucose tolerance test; HFD, high-fat diet; ITT, insulin tolerance test; LivARKO, liver androgen receptor knockout; PTT, pyruvate tolerance test; WT, wild-type.

At 2 months, the GTT trends remained the same in WT and LivARKO female mice: WT-HFD caused IGT and LivARKO prevented HFD-induced IGT (Fig. 6A and 6E, *P* < .01 orange to black and *p*<0.0001 green to orange bars, respectively). At two-months, the ITT trends remained relatively the same in WT and LivARKO female mice: WT-HFD caused IIS and LivARKO prevented HFD-induced IIS (Fig. 6B and 6F, *P* < .05 orange to black and *P* < .01 green to orange bars, respectively). At 2 months, the PTT trends changed slightly in WT and LivARKO female mice: WT-HFD and LivARKO-HFD still caused IPT (Fig. 6C and 6G, *P* < .0001 orange to black and *P* < .01 green to gray, respectively); however, LivARKO-HFD showed improvement in the IPT compared to WT-HFD (Fig. 6C and 6G, *P* < .05 green to orange). At 2 months, the GSIS trends remained the same in WT and LivARKO female mice: LivARKO-HFD and WT-HFD still caused hyperinsulinemia (HI) at 0, 15, and 30 minutes (Fig. 6D and 6H, *P* < .05 orange to black and *P* < .05 green to gray, respectively).

### Metabolic Tests in Males During Month 2

**Figure 7.**
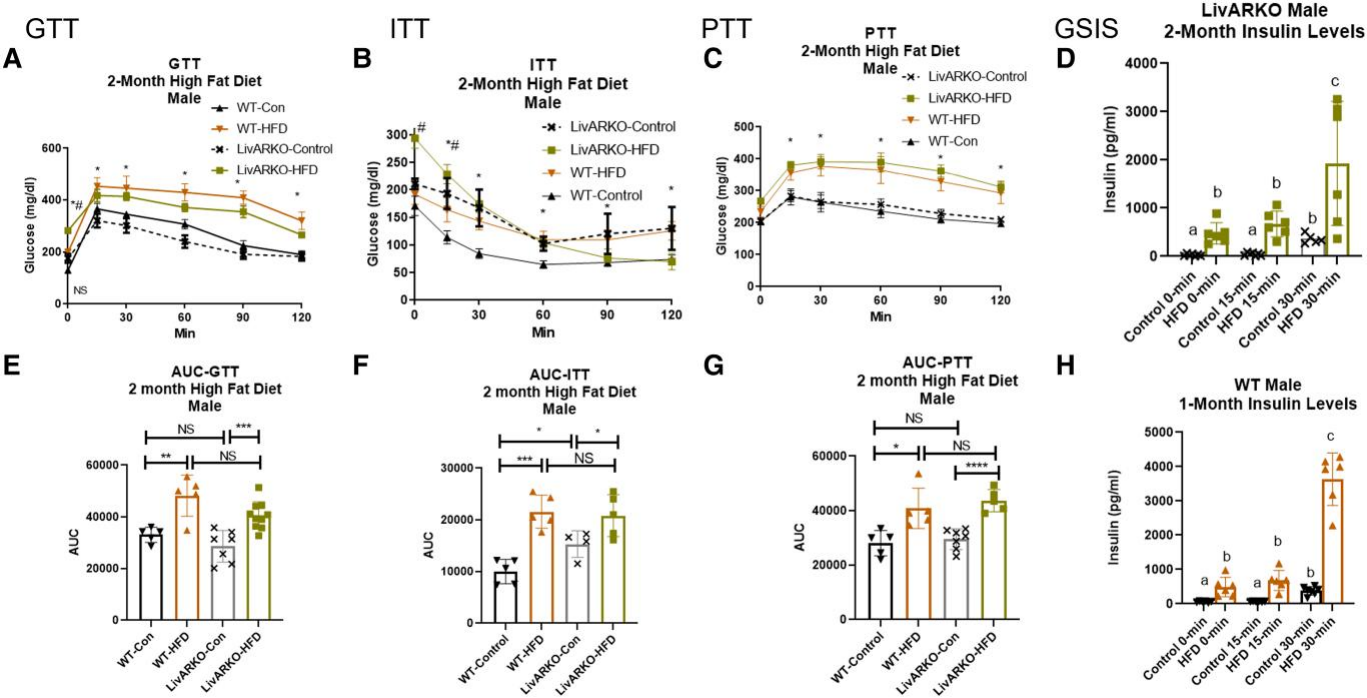
Metabolic tests in males at 2 months of HFD. During month 2 of HFD or Con diet feeding, WT and LivARKO male mice were subjected to (A) a 7-hour fasted, 2 g/kg BW intraperitoneal GTT (n = 5-8 per group), (B) a 7-hour fasted, 0.3 U/kg BW ITT (n = 5-8 per group); (C) a 7-hour fasted, 1 g/kg BW PTT (n = 5-8 per group); or (D) a 7-hour fasted GSIS analysis (LivARKO, n = 5-8 per group). (E) GTT AUC; (F) ITT AUC; (G) PTT AUC; (H) a 7-hour fasted GSIS analysis (WT, n = 5-8 per group). Difference between WT-Con and WT-HFD: *P < .05, **P < .001, ***P < .0001; difference between WT-HFD and LivARKO-HFD: #*P* < .05. Data are represented as mean ± SEM. Abbreviations: AUC, area under the curve; BW, body weight; Con, control; GSIS, glucose-stimulate; insulin secretion; GTT, glucose tolerance test; HFD, high-fat diet; ITT, insulin tolerance test; LivARKO, liver androgen receptor knockout; PTT, pyruvate tolerance test; WT, wild-type.

At 2 months, the GTT, ITT, PTT, and GSIS trends remained relatively the same in WT and LivARKO male mice: WT-HFD and LivARKO-HFD still caused IGT (Fig. 7A and 7E, *P* < .01 orange to black and *P* < .001 green to gray, respectively), IIS (Fig. 7B and 7F, *P* < .001 orange to black and *P* < .05 green to gray, respectively), IPT (Fig. 7C and 7G, *P* < .05 orange to black and *P* < .0001 green to gray, respectively), and HI (Fig. 7H and 7D, *P* < .05 orange to black and *P* < .05 green to gray, respectively). Unlike in females, LivARKO did not prevent HFD-induced metabolic dysfunction.

### Blood Glucose, Insulin and Weight in Females at 1 Month and 2 Months

**Figure 8.**
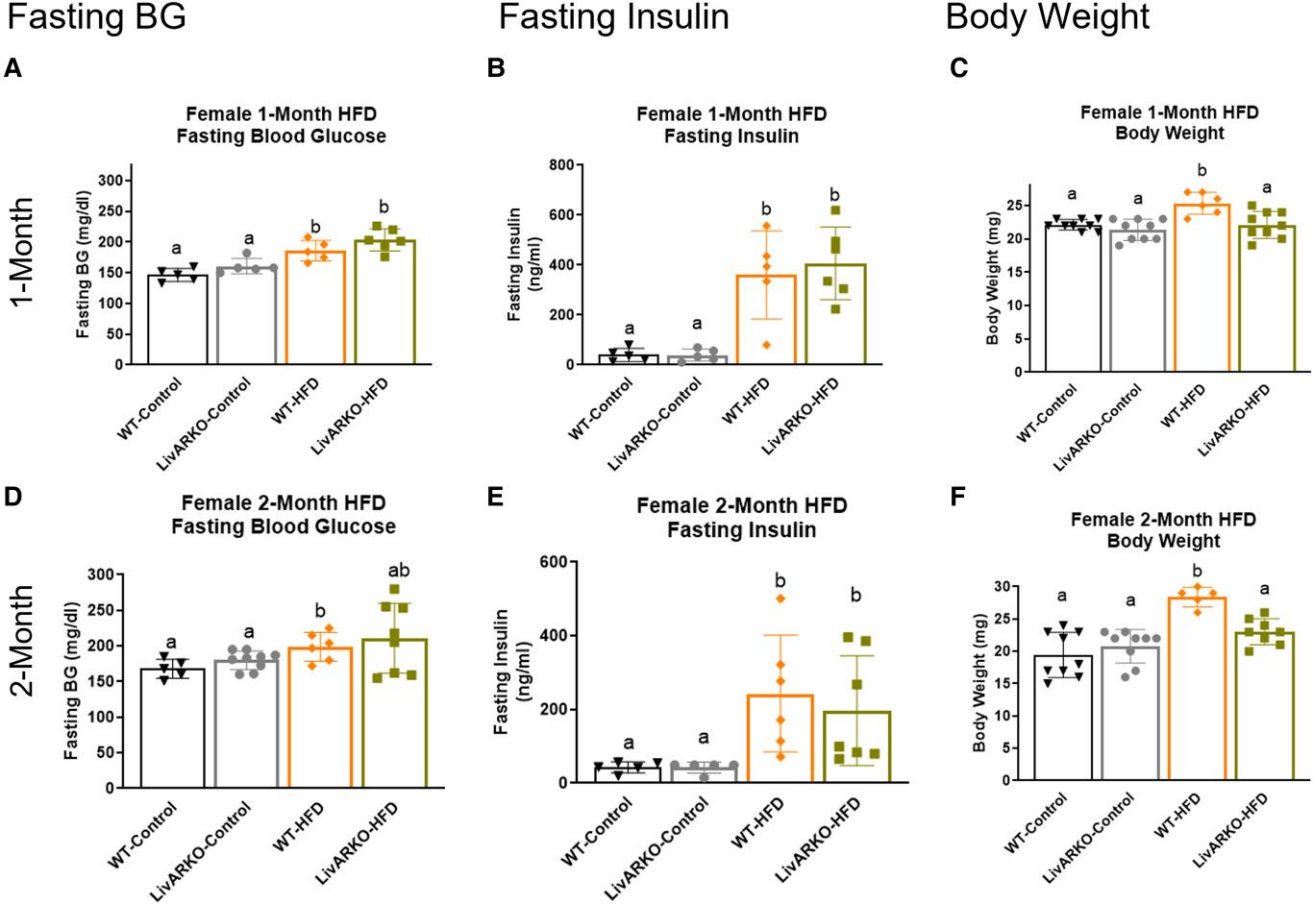
Blood glucose, insulin, and weight in females at 1 month and 2 months. (A) Fasting blood glucose levels were measured using a glucometer in WT and LivARKO female mice during months 1 and 2 of control diet and HFD feeding (n = 5-8 per group). (B) Tail-vein blood samples were obtained during months 1 and 2 of HFD or control diet feeding from WT and LivARKO female mice fasted for 7 hours, and basal insulin levels were measured by ELISA assay (n = 5-8 per group). (C) WT and LivARKO female mice fasted for 7 hours were weighed during months 1 and 2 of HFD or chow feeding (n = 5 to 7 per group). Different letters indicate *P* < .05. Abbreviations: HFD, high-fat diet; LivARKO, liver androgen receptor knockout; WT, wild-type.

HFD increased fasting blood glucose (FBG) levels in WT and LivARKO female mice at 1 month compared to controls (Fig. 8A, 1-month graph, *P* < .05, orange to black and green to gray, respectively). Interestingly, at 2 months, LivARKO-HFD FBG levels were not significantly higher than LivARKO-Con or WT-Con (Fig. 8D, 2-month graph, green to gray and green to black, respectively). HFD increased fasting insulin (FI) levels in WT and LivARKO female mice by 3-fold at 1 month (Fig. 8B, 1-month graph, *P* < .05, orange to black and green to gray, respectively) and by 2-fold at 2 months compared to controls (Fig. 8E, 2-month graph, *P* < .05, orange to black and green to gray, respectively). HFD caused a significant increase in BW in WT female mice at 1 month and 2 months (Fig. 8C and 8F, *P* < .05, 1-month and 2-months graph, orange compared to black). This increase in BW was prevented in LivARKO female mice (Fig. 8C and 8F, 1-month and 2-months graph, green compared to gray); and LivARKO-HFD displayed lower BW than WT-HFD female mice (Fig. 8C and 8F, *P* < .05, 1-month and 2-months graph, green compared to orange).

### Blood Glucose, Insulin and Weight in Males at 1 Month and 2 Months

**Figure 9.**
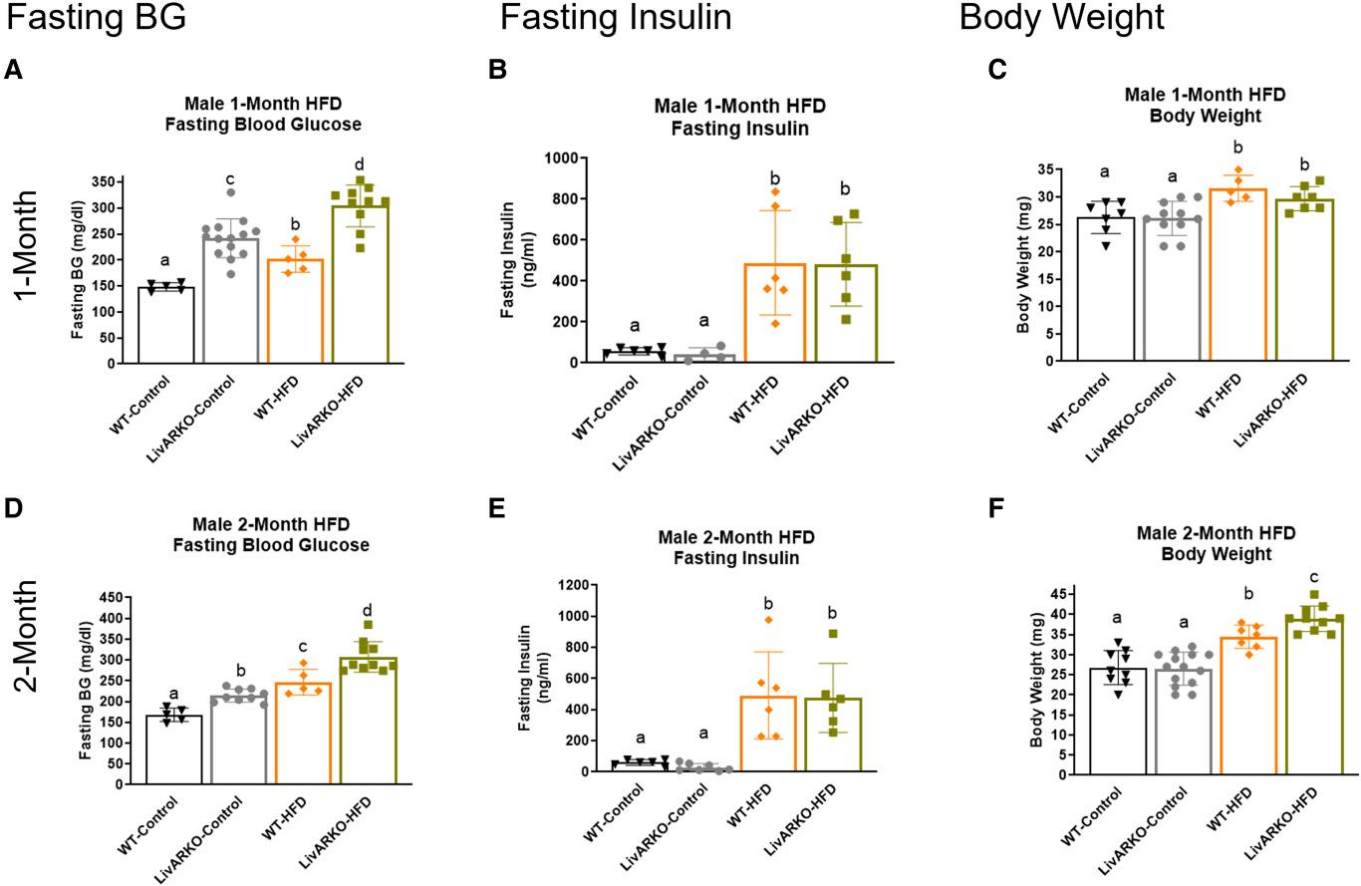
Blood glucose, insulin, and weight in males at 1 month and 2 months. (A) Fasting blood glucose levels were measured using a glucometer in WT and LivARKO male mice during months 1 and 2 of control diet and HFD feeding (n = 5-8 per group). (B) Tail-vein blood samples were obtained during months 1 and 2 of HFD or control diet feeding from WT and LivARKO male mice fasted for 7 hours, and basal insulin levels were measured by ELISA assay (n = 5-8 per group). (C) WT and LivARKO male mice fasted for 7 hours were weighed during months 1 and 2 of HFD or chow feeding (n = 5 to 7 per group). Different letters indicate *P* < .05. Abbreviations: HFD, high-fat diet; LivARKO, liver androgen receptor knockout; WT, wild-type.

Interestingly, in male mice, LivARKO-Con displayed significantly higher FBG levels than WT-Con (Fig. 9A, *P* < .05, gray compared to black bars). HFD increased FBG in WT male mice (Fig. 9A and 9D, *P* < .05, 1-month and 2-months graphs, orange to black), and this increase was exacerbated in LivARKO-HFD male mice (Fig. 9A and 9D, *P* < .05, 1-month and2-months graphs, green to orange). HFD caused significantly increased FI levels in WT and LivARKO male mice by 5-fold at 1 month (Fig. 9B, 1-month graph, *P* < .05, orange to black and green to gray, respectively) and at 2 months compared to controls (Fig. 9E, 2-months graph, *P* < .05, orange to black and green to gray, respectively). HFD significantly increased BW in WT and LivARKO male mice at 1 month and 2 months (Fig. 9C and 9F, *P* < .05, orange to black and green to gray, respectively). Interestingly, at 2 months, LivARKO exacerbated the increased BW as LivARKO-HFD was higher than WT-HFD (Fig. 9F, *P* < .05, 2-months graph, green to orange).

### Plasma Estradiol , Liver *Esr1* mRNA Levels, and Liver ERα Protein Expression in Control vs HFD LivARKO Mice

**Figure 10.**
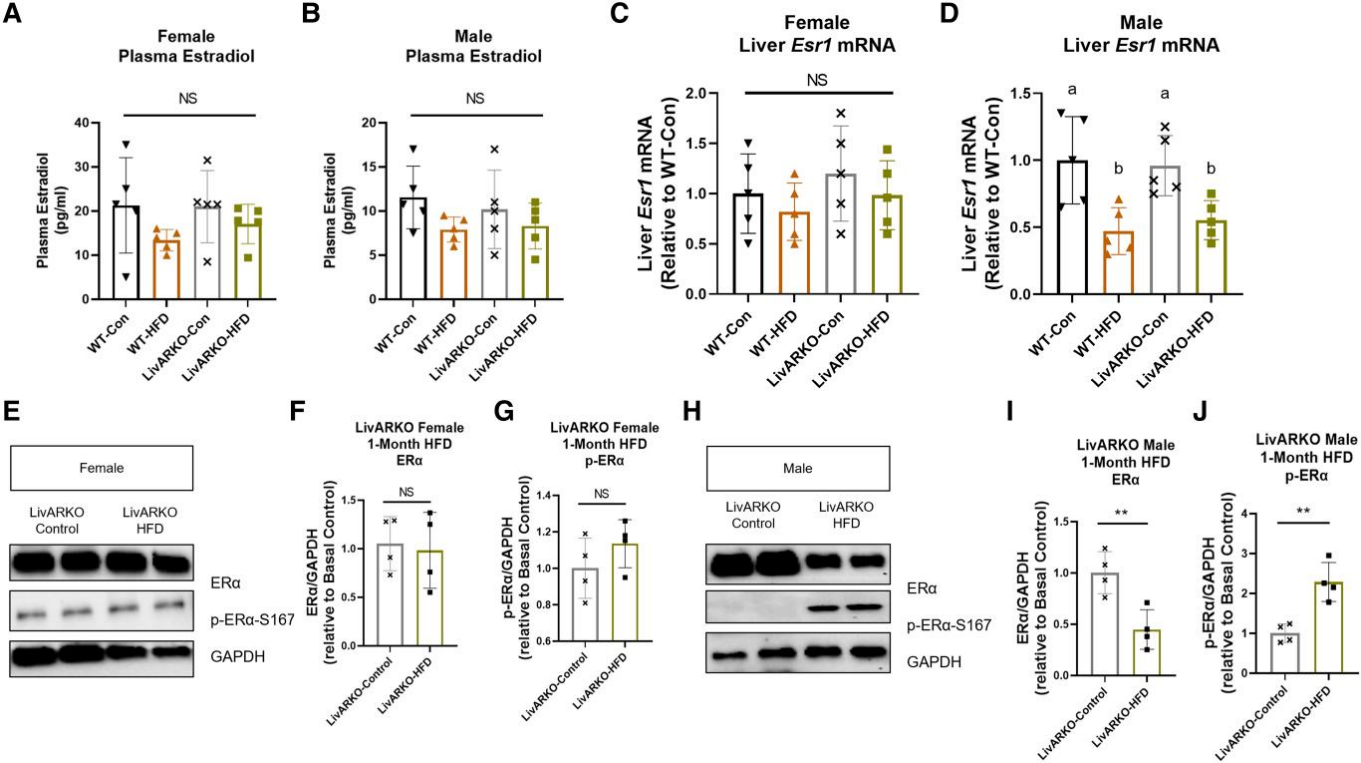
Plasma estradiol, liver *Esr1* mRNA levels, and liver ERα protein expression in control vs HFD LivARKO mice. WT and LivARKO mice fed a Con or HFD for 1 month were examined for the following: (A) plasma estradiol levels in female mice (n = 5 per group); (B) plasma estradiol levels in male mice (n = 5 per group). WT and LivARKO mice fed a Con diet or HFD for 1 month were examined for the following: (C) liver *Esr1* mRNA expression in female mice (n = 5 per group); (D) liver *Esr1* mRNA expression in male mice (n = 5 per group). LivARKO mice fed a Con diet or HFD for 1 month were examined for the following: (E-G) ERα and p-ERα-S167 protein expression in female mice (n = 4 per group); (H-J) ERα and p-ERα-S167 protein expression in male mice (n = 4 per group). Different letters indicate P < .05. ** *P* < .01. Abbreviations: Con, control; Erα, estrogen receptor alpha; HFD, high-fat diet; LivARKO, liver androgen receptor knockout; WT, wild-type.

Serum estrogen levels were measured in male and female WT and LivARKO mice. Female mice displayed similar estrogen levels in all of the groups measured (Fig. 10A). Similarly, in male mice, HFD did not alter estrogen levels in WT or LivARKO mice compared to male mice on the control diet (Fig. 10B). Liver *Esr1* mRNA and ERα protein levels were measured in male and female LivARKO mice. Female LivARKO mice on control diet or HFD displayed no change in *Esr1* mRNA expression (Fig. 10C) or ERα protein levels (Fig. 10E and 10F) in comparison to each other. However, male LivARKO-HFD mice displayed lower *Esr1* mRNA expression (Fig. 10D) and lowered ERα protein levels (Fig. 10H and 10I) compared to male LivARKO-Con mice. Additionally, p-Erα-S167 expression was significantly higher in LivARKO-HFD male mice compared to Con-LivARKO male mice (Fig. 10H and 10J).

### Insulin-Stimulated Liver p-AKT Protein Expression and Gluconeogenic mRNA Expression in Control vs HFD LivARKO Mice

**Figure 11.**
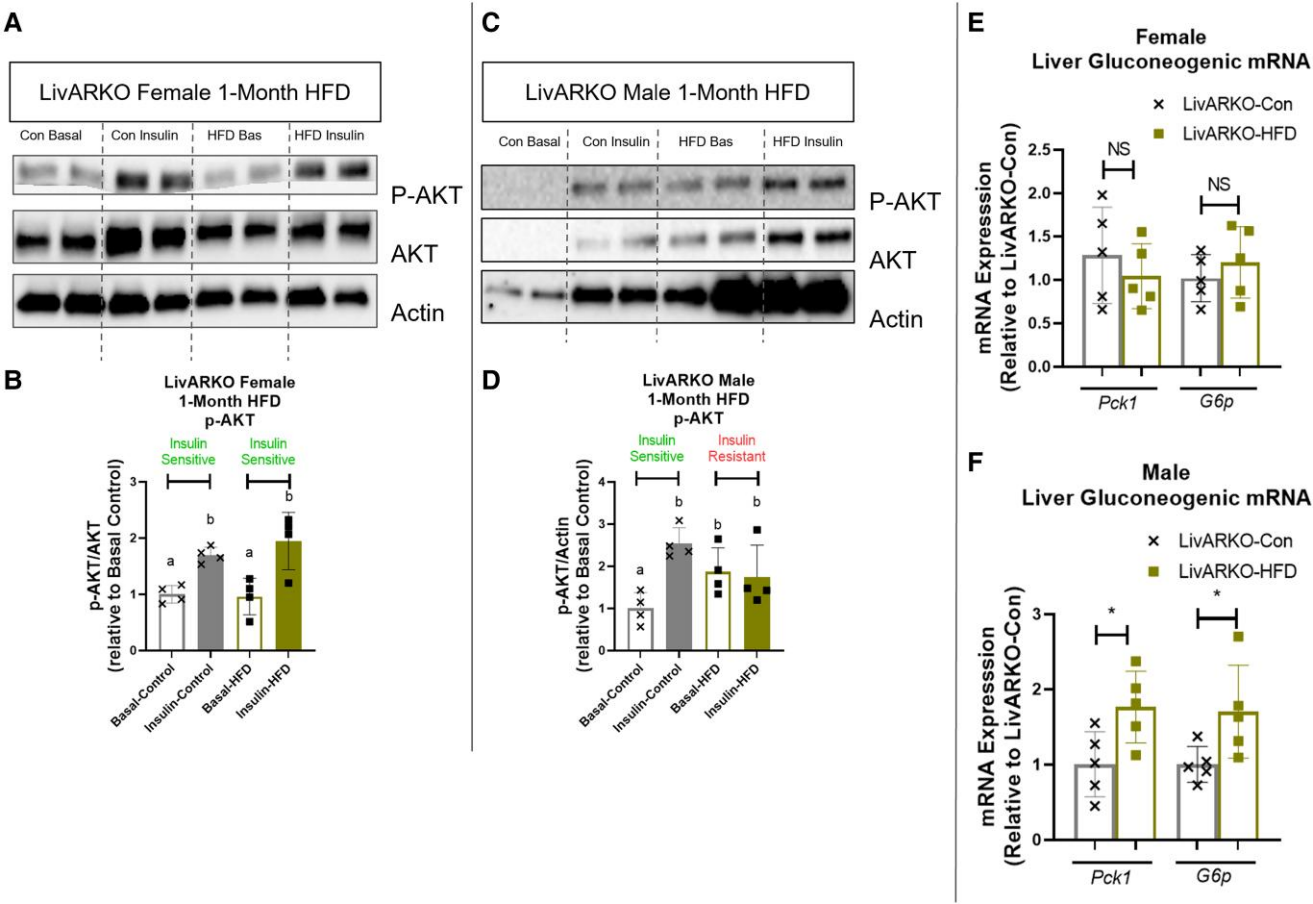
Insulin stimulated liver p-AKT (Ser473) protein expression and gluconeogenic mRNA expression in control vs HFD LivARKO mice. LivARKO mice fed a Con diet or HFD for 1 month were examined for the following: (A) insulin-stimulated liver p-AKT (Ser473) and AKT protein expression in female mice (n = 4 per group); (B) insulin-stimulated liver p-AKT (Ser473) and AKT protein expression in male mice (n = 4 per group); (C) gluconeogenic mRNA expression (*Pck1* and *G6p*) in female mice (n = 5 per group); (D) gluconeogenic mRNA expression (*Pck1* and *G6p*) in male mice (n = 5 per group). Different letters indicate *P* < .05. * *P* < .05. Abbreviations: Con, control; *G6p*, glucose 6-phosphate; HFD, high-fat diet; LivARKO, liver androgen receptor knockout; *Pck1*, phosphoenolpyruvate carboxykinase 1.

To assess the function of insulin action in the liver, p-AKT (Ser473), AKT, and actin protein expression in liver samples of Con and HFD LivARKO female and male mice was evaluated. Insulin increased p-AKT (S473) in the livers of LivARKO-Con female and male mice compared to mice not given insulin (Fig. 11A and 11D, gray unfilled compared to gray filled bars, *P* < .05). Female LivARKO-HFD mice remained insulin sensitive displaying increased p-AKT (Ser473) in the liver compared to female LivARKO-HFD mice not given insulin (Fig. 11A and 11B, green unfilled compared to green filled bars, *P* < .05). However, male LivARKO-HFD mice displayed lowered insulin stimulated p-AKT in the liver compared to mice not given insulin (Fig. 11C and 11D, green unfilled compared to green filled bars), suggesting that these mice are experiencing hepatic insulin resistance.

To determine the levels of gluconeogenic mRNA expression, livers from Con and HFD LivARKO female and male mice were evaluated via quantitative real-time PCR analysis for expression of *Pck1* and *G6p* mRNA expression. *Pck1* and *G6p* mRNA expression in the liver was unaltered in female LivARKO-Con compared to LivARKO-HFD mice (Fig. 11E). However, in male mice, *Pck1* and *G6p* mRNA expression in the liver was increased in LivARKO-HFD male mice compared to LivARKO-Con male mice (Fig. 11F, *P* < .05).

## Discussion

We hypothesized that the deletion of hepatic AR would play a minimal role in HFD-induced metabolic defects (IGT, IIS, IPT, and HI) in female mice. We anticipated this because HFD-induced insulin resistance is proposed to involve the DAG-induced activation of PKC leading to disrupted IR phosphorylation [4], whereas, HA-induced insulin resistance did not lower IR tyrosine phosphorylation [12], suggesting that the DAG-induced activation of PKC does not involve AR. Higher doses of HA and prolonged HA resulted in increased hepatic lipid content [17], which may then likely lead to increased DAG. However, how AR might play a role in lipid-induced insulin resistance is not fully understood. Our hypothesis was disproved as LivARKO played a significant role in improving HFD-induced metabolic derangements in female mice. It should also be noted that DAG-induced increased PKCε as a mechanism of reduced insulin receptor phosphorylation has been challenged as not being a mechanism of hepatic insulin resistance [24].

Collectively, the 1-month metabolic tests in females suggested that LivARKO was able to prevent HFD-induced impaired glucose tolerance and improved insulin sensitivity. However, LivARKO did not prevent HFD-induced HI, and it did not prevent HFD-induced impaired pyruvate tolerance in female mice. In contrast, LivARKO was able to prevent high androgen-induced IPT and HI [11]. Unlike in females, LivARKO in males was not protective or preventative regarding glucose metabolism. In males, LivARKO did not prevent HFD-induced IGT or IPT. Most notably, unlike in females, male LivARKO-Con mice displayed worse glucose, insulin, and pyruvate tolerance compared to WT-Con male mice, suggesting that removing AR in liver of males was detrimental even without a HFD challenge. The improvements that began at 1 month in LivARKO female mice persisted at 2 months. The phenotype present in LivARKO male mice at 1 month remained relatively the same at 2 months. In comparison to low-dose high androgens [11], which did not increase the weight of WT female mice, these data show that LivARKO prevented HFD-induced weight gain in female mice. In contrast, as seen in Lin et al 2008 [10], in male mice, LivARKO exacerbated HFD-induced weight gain.

Previous studies have shown that after 16 to 20 weeks of HFD feeding, mice will display a roughly 20% to 30% higher BW compared to chow-fed mice [25]. Here, we see a 20% to 25% increase in HFD-induced BW after 8 weeks of HFD in WT-Con compared to WT-HFD male and female mice (Figs. 8C and 9C). Interestingly, LivARKO prevented this BW gain in females but exacerbated the weight gain in males after 8 weeks of HFD. The hyperglycemic phenotype has been shown within 4 weeks of HFD [26], and here we see that same trend in female and male mice (Figs. 8A and 9A; increased fasting blood glucose in WT-HFD compared to WT-Con). LivARKO exacerbated the hyperglycemia in males and failed to prevent the hyperglycemia in females. Higher fasting glucose levels are typically accompanied by higher fasting insulin levels. Here we see higher fasting insulin levels in WT-HFD compared to WT-Con in males and females (Figs. 8B and 9B). Interestingly, LivARKO did not exacerbate or prevent HI in male or female mice.

Intriguingly, although LivARKO-HFD female mice do not display impaired glucose or insulin tolerance, they do still exhibit HI. This may be because LivARKO improves hepatic insulin signaling (Fig. 11A) but may not improve skeletal muscle or WAT insulin signaling (findings yet to be examined). With skeletal muscles and WAT still experiencing HFD-induced insulin resistance, this may still trigger the pancreatic beta cells to release insulin. One study found that in obese human subjects with normal glycemic levels, higher serum free fatty acids (FFA) at fasting was the primary metabolic problem propagating the fasting HI [27]. Examining serum FFA levels in this model would be a future study that could help address the phenotype of HI in the face of normal glycemia seen in LivARKO-HFD female mice. Another study observed that beta cell proliferation was significantly elevated after only 3 days of HFD feeding, and this occurred weeks before peripheral insulin resistance was present [28]. Thus, alternatively, HFD could be directly altering or increasing the pancreatic beta cell release of insulin in LivARKO-HFD female mice, where deletion of AR in the liver has no impact on the higher release of insulin from the pancreatic beta cells. Examining beta cell function in this model in future studies is warranted.

The ER function is known to be protective against diet-induced metabolic dysfunction [29]. Previous studies have shown that *Esr1* mRNA and ERα protein levels in energy storage tissues of WT female mice on a chow diet or a HFD were unaltered [30]. Similarly, here we show that female HFD-LivARKO mice displayed no change in *Esr1* mRNA and ERα protein levels compared to Con-LivARKO female mice. However, male HFD-LivARKO mice had lowered *Esr1* mRNA, lowered ERα protein, and increased p-Era-S167 compared to male Con-LivARKO mice. ERα Ser167 may be phosphorylated by Akt [31, 32]. The HFD-induced lowered ER mRNA and protein levels in the livers of WT and LivARKO male mice likely contributed to the whole-body glucose dysregulation. However, deletion of liver AR in male mice did not appear to improve or exacerbate this HFD-induced lowered ER phenomenon.

Lin et al [10] was the first study to place LivARKO male and female mice on an HFD. Some notable differences between this study and the Lin 2008 study were the following. For the GTT tests, FBG, and FI in the Lin et al 2008 study, the mice were fasted for 14 hours and here they were fasted for 7 hours. The male LivARKO-HFD mice displayed exacerbated IGT compared to WT-HFD in the Lin et al 2008 study. Here, the male LivARKO-HFD mice displayed IGT, but it was not exacerbated in comparison to WT-HFD. The Lin et al 2008 study found no difference in FBG in male WT-Con compared to LivARKO-Con, but here LivARKO increased FBG in males. This may be a result of the different fasting times, where LivARKO may be impacting liver glycogenolysis, whereas at a 7-hour fast glycogen is not fully depleted [20]. Additionally, HFD did not significantly increase FBG in WT male or female mice in the Lin et al 2008 study, but HFD did increase FBG (resulting in hyperglycemia) in our study. This may be due to the percentage of fat in the respective HFD. This study used 60%, and here we used a calorie-matched control diet instead of chow. The percentage of fat was not reported in the Lin et al 2008 study. For the ITT tests, the mice were injected with 0.3 units/kg BW insulin in this study as opposed to 1 units/kg BW in the Lin et al 2008 study. Here, LivARKO-HFD had a lower BW than WT-HFD at 2 months on the HFD, whereas, in the Lin et al 2008 study, LivARKO-HFD and WT-HFD had no change in BW at 2 months on the HFD.

Regarding limitations and things to consider, it should be noted that HFD feeding is often initiated at 6 to 10 weeks of age when using diet-induced mouse models to achieve obesity and/or glucose metabolic dysfunction [15]. Some studies have shown that starting a HFD during the peripubertal period (4 weeks of age) may lessen the degree of metabolic dysfunction in comparison to starting the HFD during adult age (6-10 weeks of age) [15]. However, although the degree of dysfunction is lessened when started during the peripubertal age, mice started on a HFD at the peripubertal age still developed glucose metabolic dysfunction in comparison to mice on a low-fat chow diet [16]. Thus, if the goal is to assess metabolic challenges starting during the peripubertal age, as was the case for this study, then starting a HFD at 4 weeks of age is feasible.

Previous studies [33] and here showed that HFD feeding resulted in weight gain in female mice. It is interesting to note that the LivARKO female mice did not gain weight upon HFD feeding. Activity and food intake would be interesting to examine in future studies. An interesting question is: Where are the excess calories going? Do the HFD-LivARKO female mice exhibit a fatty liver phenotype? Do the HFD-LivARKO female mice display elevated serum triglycerides or free fatty acids? Determining liver lipid content, serum triglycerides, and/or serum free fatty acids would add great insight. As mentioned earlier, examining the serum free fatty acid levels could help explain the HI phenotype in LivARKO-HFD female mice. With this study focusing on glucose metabolism, the authors felt that examining lipid metabolism was outside of the scope of this manuscript.

Deletion of hepatic ARs in mice (LivARKO) may lead to changes in other hormones, such as sex hormones and others. Recent publications using the LivARKO mouse model have shown that LH and FSH levels were unaltered [34] and that C-peptide levels were unchanged [11]. In this study, we observed that HFD and LivARKO did not have an impact on serum estradiol levels. Measuring the levels of other hormones would be beneficial. Future studies could benefit from performing these studies in ovariectomized female mice to further elucidate if any crosstalk between ER and AR exist in this context.

Previous studies have shown dimorphic effects of HFD feeding on glucose metabolism. HFD and sex are strong modulators of metabolic readouts in C57BL/6N mice [35]. Considering that dimorphic effects are present, it has been documented that diabetes research has been stifled by the underrepresentation of females in animal and human studies [36]. A recent study showed that male C57BL/6 mice developed more diet-induced weight gain and that a HFD lowered activity levels in males but not females [37]. In this study, female mice displayed protection from developing diet-induced insulin resistance. Additionally, beta cell mass was enlarged in males in response to an HFD. The mechanisms for why we observed the dimorphic effects we observed in this study warrant further investigation. Taken together, these data show that ARs display dimorphic effects in the liver regarding regulation of whole-body glucose metabolism. Where deletion of AR in the liver of female mice prevented multiple readouts of HFD-induced dysglycemia; deletion of AR in the livers of male mice exacerbated several features of HFD-induced dysglycemia. Future studies examining the molecular mechanisms of these dimorphic effects are warranted.

## Data Availability

Some or all datasets generated during and/or analyzed during the current study are not publicly available but are available from the corresponding author on reasonable request.

## Abbreviations

AR: androgen receptor
AUC: area under the curve
BW: body weight
Con: control
DAG: diacylglycerol
ER: estrogen receptor
FBG: fasting blood glucose
FI: fasting insulin
*G6p*: glucose 6-phosphate
GSIS: glucose-stimulated insulin secretion
GTT: glucose tolerance test
HFD: high-fat diet
HA: hyperandrogenemia
HI: hyperinsulinemia
IGT: impaired glucose tolerance
IIS: impaired insulin sensitivity
IPT: impaired pyruvate tolerance
IR: insulin receptor
ITT: insulin tolerance test
LivARKO: liver androgen receptor knockout
*Pck1*: phosphoenolpyruvate carboxykinase 1
PKC: protein kinase C
PKCε: protein kinase C epsilon
PTT: pyruvate tolerance test
WAT: white adipose tissues
